# A novel Aβ isoform pattern in CSF reflects γ-secretase inhibition in Alzheimer disease

**DOI:** 10.1186/alzrt30

**Published:** 2010-03-29

**Authors:** Erik Portelius, Robert A Dean, Mikael K Gustavsson, Ulf Andreasson, Henrik Zetterberg, Eric Siemers, Kaj Blennow

**Affiliations:** 1 Department of Psychiatry and Neurochemistry, Institute of Neuroscience and Physiology, The Sahlgrenska Academy at the University of Gothenburg, Molndal, SE-431 80, Sweden; 2 Translational Medicine, Eli Lilly and Company, Lilly Corporate Headquarters, Indianapolis, IN 46285, USA; 3 Global Alzheimer's Disease Research Team, Eli Lilly and Company, Lilly Corporate Headquarters, Indianapolis, IN 46285, USA

## Abstract

**Introduction:**

LY450139 (semagacestat) inhibits γ-secretase, a key enzyme for generation of amyloid β (Aβ), the peptide deposited in plaques in Alzheimer disease (AD). Previous data have shown that LY450139 lowers plasma Aβ, but has no clear effect on Aβ1-40 or Aβ1-42 levels in cerebrospinal fluid (CSF). By using targeted proteomics techniques, we recently identified several shorter Aβ isoforms, such as Aβ1-16, that in experimental settings increase during γ-secretase inhibitor treatment, and thus may serve as sensitive biochemical indices of the treatment effect. Here, we test the hypothesis that these shorter Aβ isoforms may be biomarkers of γ-secretase inhibitor treatment in clinical trials.

**Methods:**

In a phase II clinical trial, 35 individuals with mild to moderate AD were randomized to placebo (*n* = 10) or LY450139 (100 mg (*n* = 15) or 140 mg (*n* = 10)) and underwent lumbar puncture at baseline and after 14 weeks of treatment. The CSF Aβ isoform pattern was analyzed with immunoprecipitation combined with MALDI-TOF mass spectrometry.

**Results:**

The CSF levels of Aβ1-14, Aβ1-15, and Aβ1-16 showed a dose-dependent increase by 57% and 74%, 21% and 35%, and 30% and 67%, respectively in the 100-mg and 140-mg treatment groups. Aβ1-40 and Aβ1-42 were unaffected by treatment.

**Conclusions:**

CSF Aβ1-14, Aβ1-15, and Aβ1-16 increase during γ-secretase inhibitor treatment in AD, even at doses that do not affect Aβ1-42 or Aβ1-40, probably because of increased substrate availability of the C99 APP stub (APP β-CTF) induced by γ-secretase inhibition. These Aβ isoforms may be novel sensitive biomarkers to monitor the biochemical effect in clinical trials.

**Trial registration:**

Clinical Trials.gov NCT00244322

## Introduction

**Figure 1:**
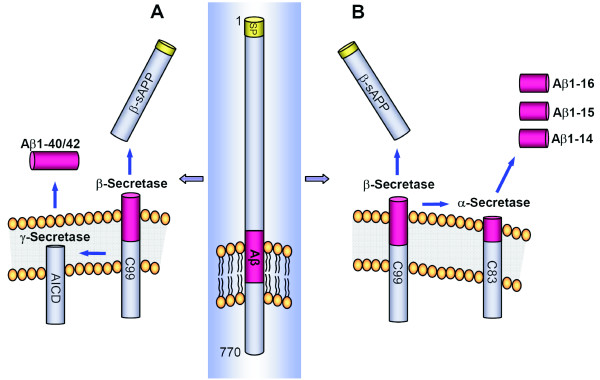
**Schematic drawing of APP and generation of Aβ isoforms**. The 17-amino acid signal peptide is indicated at the N-terminus. A single membrane-spanning domain is located at amino acids 700-723 in the longest APP isoform (APP770). **(a)** In the amyloidogenic pathway, β-secretase cleaves after residue 671, generating β-sAPP, which is secreted, and a C-terminal fragment (β-CTF or C99), which is retained in the membrane. The β-CTF fragment can undergo further cleavage by γ-secretase to release Aβ isoforms. **(b)** In another pathway, APP is first cleaved by β-secretase, but after this, by α-secretase, thus generating the shorter isoforms Aβ1-14, Aβ1-15, and Aβ-16. In another described nonamyloidogenic pathway, α-secretase cleaves between amino acids 16 and 17 in the Aβ sequence generating α-sAPP, followed by γ-secretase cleavages, generating a fragment called p3 (Aβ17-40/42). This 3-kDa fragment has been isolated from cell-culture medium [29] and in brains from AD patients [30]. However, the fragment has never been detected in human CSF. AICD, APP intracellular domain; APP, amyloid precursor protein; Aβ, amyloid β; sAPP, soluble amyloid precursor protein.

Accumulation of amyloid β (Aβ) peptides in senile plaques in the cerebral cortex is an early event in the pathogenesis of Alzheimer disease (AD) [1]. The longest isoform of Aβ, consisting of 42 amino acids (Aβ1-42), is produced from amyloid precursor protein (APP) by sequential cleavage by β- and γ-secretase in the amyloidogenic APP-processing pathway (Figure 1A) [2]. β-Secretase activity originates from an integral membrane aspartyl protease encoded by the β-site APP-cleaving enzyme 1 gene (*BACE1*) [3-6] whereas γ-secretase is an intramembrane-cleaving complex composed of at least four essential subunits with the presenilin (PS1 or PS2) proteins at its enzymatic core [7-9]. γ-Secretase, which is one of the top targets for developing AD therapeutics with disease-modifying effects, cleaves the transmembrane region of APP to produce Aβ of variable length [10-12], with Aβ1-40 being the most abundant isoform, whereas Aβ1-42 is most prone to aggregation [13,14].

The main focus of using disease-modifying drugs is to inhibit brain Aβ production and aggregation and to increase Aβ clearance from the brain. Several drug candidates, including β-secretase and γ-secretase inhibitors or Aβ immunotherapy, are being evaluated in different phases in clinical trials. However, in a slowly progressive disorder like AD, evaluation of the clinical effect of a disease-modifying drug by using rating scales requires large patient numbers and extended treatment periods. This type of drug cannot be expected to have any early effect on symptoms, but instead may show a less-pronounced decline in cognitive function over years. Thus, a great need exists for biomarkers to identify and monitor the biochemical effect of disease-modifying drugs in AD clinical trials. Such biomarker data providing evidence directly in patients with AD that the drug has the predicted mechanism of action may be valuable for making a go/no-go decision for large and expensive Phase II or III clinical trials.

In animal studies, a decrease in soluble Aβ levels and Aβ accumulation has been reported in response to γ-secretase inhibition [15-18]. Recently, in a human clinical trial, it was shown, by using enzyme-linked immunosorbent assay (ELISA), that the γ-secretase inhibitor LY450139 reduces the concentration of Aβ1-40 and Aβ1-42 in human plasma in a dose-dependent manner, whereas a significant decrease in human cerebrospinal fluid (CSF) Aβ levels was not detected [19]. However, by using a stable isotope-labeling kinetic (SILK) method, which can be used to measure central nervous system (CNS) production and clearance rates of total Aβ in the CSF of humans [20], it was shown that LY450139 significantly decreased Aβ production without affecting Aβ clearance in the CNS [21].

We recently identified a set of 18 N- and C-terminally truncated Aβ peptides in human CSF by using immunoprecipitation-mass spectrometry (IP-MS) and showed that CSF Aβ1-16 may be a novel positive biomarker for AD that was elevated in AD patients [22]. This novel biomarker for AD is generated through a previously unrecognized metabolic pathway by concerted β- and α-secretase cleavage of APP (Figure 1B) [23]. Further, the relative level of Aβ1-16, along with Aβ1-14 and Aβ1-15, has previously been shown to be elevated in cell media and in CSF from mice in response to γ-secretase inhibitor treatment [23,24], suggesting that these shorter Aβ isoforms may be sensitive biomarkers for γ-secretase inhibitor treatment. Here, we tested the hypothesis that treatment of AD patients with the γ-secretase inhibitor LY450139 can be monitored by increased CSF levels of Aβ1-14, Aβ1-15, and Aβ1-16.

## Materials and methods

### Study participants and sample collection

**Figure 2:**
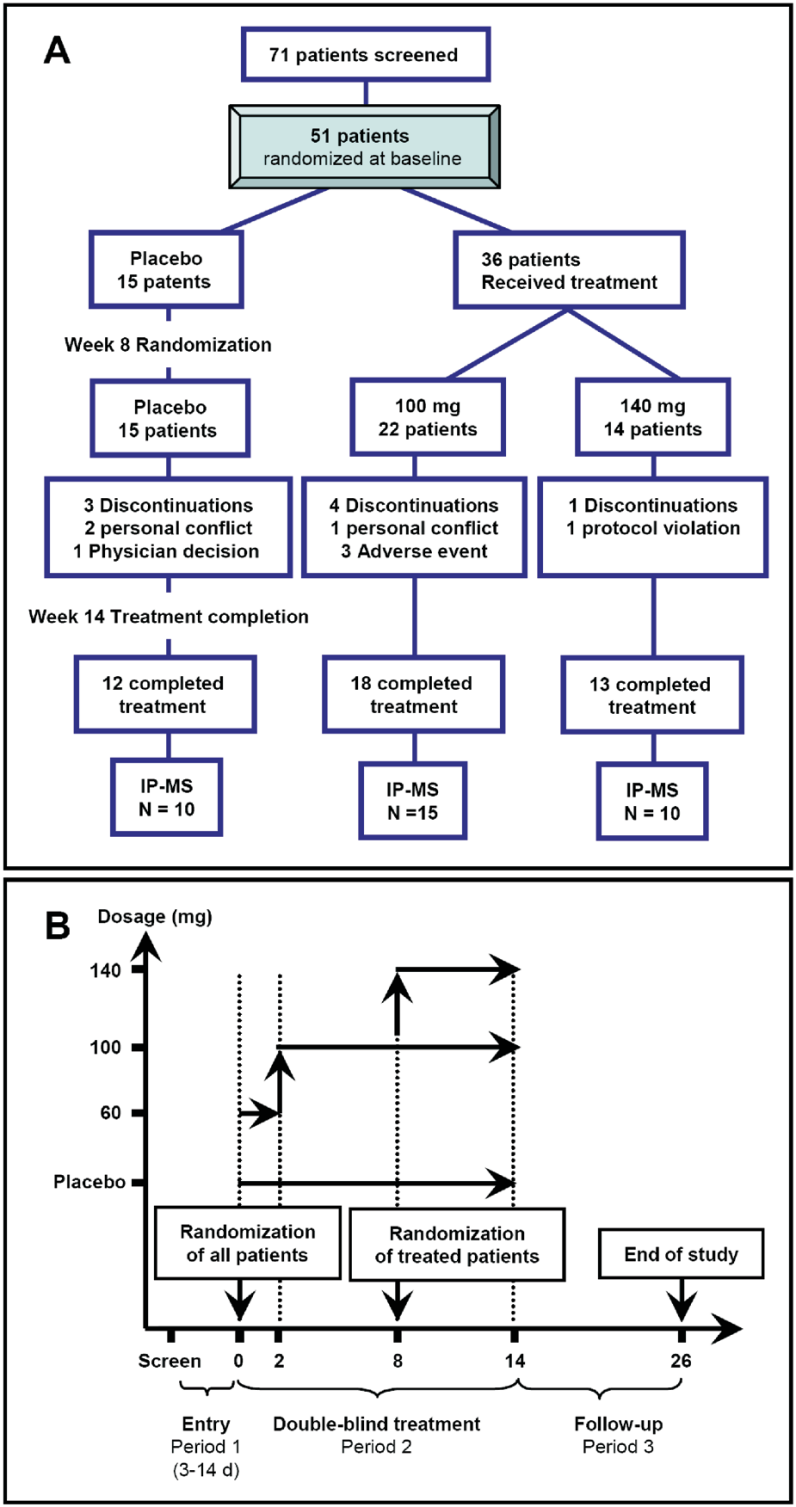
**Data flowchart for the trial**. **(a)** The intention-to-treat analyses included all 51 patients originally randomized, including all subsequent dropouts. Of the 51 patients, 35 were analyzed by using IP-MS. **(b) **Study design.

The study cohort and clinical procedures have been described before in detail [19]. In brief, 51 patients were enrolled at six academic research centers between October 1, 2005, and December 31, 2006 (Figure 2a). The protocol was reviewed and approved by the institutional review board at each participating site. All research participants and caregivers gave written informed consent. Of the 51 patients enrolled in the study, CSF samples from 35 patients were available for analysis by IP-MS. See Table 1 for demographic data.

**Table 1:** Participant characteristics at baseline

	Placebo	LY450139, 100 mg	LY450139, 140 mg
Characteristic	(*n* = 10)	(*n* = 15)	(*n* = 10)
Demographics			
Age, mean (SD, years)	69.1 (8.81)	72.4 (8.13)	68.8 (9.02)
Female sex, number (%)	5 (50)	10 (66.6)	4 (40)
APOE e4 carriers, number (%)	8 of 10 (80)	11 of 13 (84.6)	7 of 9 (77.8)
Clinical scores, mean (SD)			
MMSE	18.3 (4.0)	22 (3.6)	24.9 (1.4)
ADCS-ADL	61.9 (7.3)	67.7 (8.4)	70.6 (7.8)
ADAS-Cog 11	26.9 (10.0)	19.9 (6.3)	17.9 (5.8)

ADAS-Cog 11, Alzheimer's Disease Assessment Scale Cognitive Subscale; ADCS-ADL, Alzheimer's Disease Cooperative Study Activities of Daily Living Scale; APOE, apolipoprotein E; MMSE, Mini-Mental State Examination.

Participants were 50 years or older and diagnosed as having probable AD, as defined by the National Institute of Neurological and Communicative Disorders and Stroke and the Alzheimer Disease and Related Disorders Association (NINCDS-ADRDA) criteria [25]. Individuals receiving stable doses of cholinesterase-inhibitor drugs or memantine were included. The trial was a multicenter, randomized, double-blind, placebo-controlled, dose-escalation study (Figure 2a). Participants were randomized to receive LY450139 or placebo by using a 2:1 randomization scheme through a telephone-based interactive voice-response system (Figure 2b). Patients randomized to the LY450139 groups received 60 mg/d for 2 weeks, and then 100 mg/d for the next 6 weeks. At 8 weeks, the treatment arm was randomized again to receive 6 additional weeks of treatment at 100 or 140 mg/d.

The CSF samples were collected into polypropylene tubes by means of a lumbar puncture at baseline and approximately 6 hours after the administration of the last dose of LY450139 at week 14.

### APP measurements

CSF concentrations of α-secretase-cleaved soluble APP (α-sAPP) and β-secretase-cleaved soluble APP (β-sAPP) were determined by using the MSD sAPPα/sAPPβ multiplex assay, as described by the manufacturer (Meso Scale Discovery, Gaithersburg, MD, USA). This assay uses the 6E10 antibody to capture α-sAPP and a neoepitope-specific antibody to capture β-sAPP. Both isoforms are detected by the SULFO-TAG-labeled anti-APP antibody p2-1.

### IP-MS

IP was combined with matrix-assisted laser desorption/ionization time-of-flight (MALDI-TOF) MS for analyzing the Aβ isoform pattern in a single analysis. The IP-MS was conducted as described before [26]. In brief, 8 μg of the monoclonal antibody 6E10 (Aβ epitope 4-9; Signet Laboratories, Inc., Dedham, MA, USA) was added to 50 μl Dynabeads M-280 (Dynal) sheep anti-mouse and left overnight on a rocking platform (+4°C). The IP was conducted overnight (+4°C) on 940 μl CSF, to which 10 μl 2.5% Tween-20 (Bio-Rad Laboratories, Inc.) had been added. The beads/CSF solution (total volume, 1 ml) was transferred to a KingFisher magnetic particle processor (polypropylene tubes; Thermo Scientific) for washing and elution in a five-step procedure. The collected supernatant was dried in a vacuum centrifuge and redissolved in 5 μl 0.1% formic acid in 20% acetonitrile.

The samples were analyzed with MALDI-TOFMS (Autoflex, Bruker Daltonics, Bremen, Germany) operating in reflector mode. The acquired MS data were analyzed by the software Medicwave Bioinformatics Suite (MBS) by using the immunoprecipitation for MALDI (IPM) module (MedicWave, Halmstad, Sweden) for automatic processing of the all spectra. In brief, after internal calibration, baseline subtraction, and smoothing, the peaks for each spectrum were integrated with the integration limits -2 to +5 m/z relative to the monoisotopic peak. Before the statistical analysis, the peak areas were normalized to the sum of the integrated peaks, duplicated samples were averaged, and the relative changes compared with baseline values were calculated, as described previously [22].

Group differences were analyzed by using the Kruskal-Wallis test, and in case of significance (*P* < 0.05), followed by Mann-Whitney test to identify the differences.

## Results

**Figure 3:**
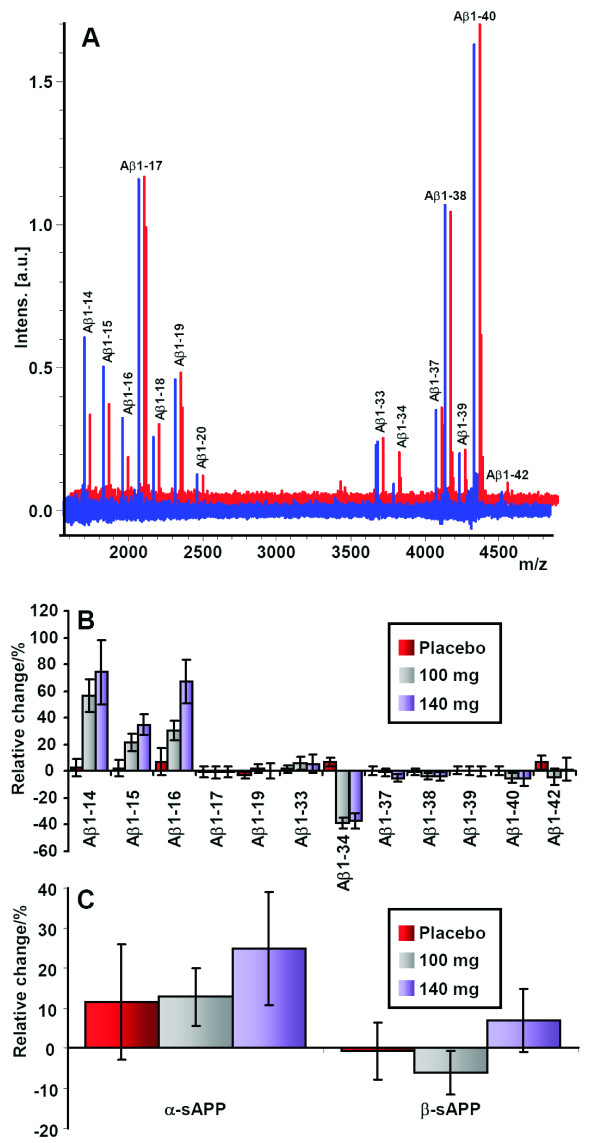
**Representative mass spectra**. **(a) **Representative mass spectra from an AD patient displaying the Aβ-isoform pattern before treatment (red spectra) and after treatment with 140 mg of the γ-secretase inhibitor LY450139 (blue spectra). The mass spectrometric signal of Aβ1-14/15/16 increased, whereas Aβ1-34 decreased. The longer isoforms, including Aβ1-17, Aβ1-40, and Aβ1-42, were unaffected by the treatment. The relative change as a response to treatment is displayed for **(b) **all Aβ isoforms detected in all samples and **(c) **α-sAPP and β-sAPP. The error bars represent standard errors of the mean.

Representative CSF Aβ isoform mass spectra from an AD patient before treatment and after 14 weeks on the high dosage of the γ-secretase inhibitor LY450139 are shown in Figure 3a. The treatment greatly increased the mass spectrometric signal for Aβ1-14, Aβ1-15, and Aβ1-16, whereas the mass spectrometric signal corresponding to Aβ1-34 decreased as a response to treatment (Kruskal-Wallis test, *P *= 0.009, 0.01, 0.05, and 0.000041, respectively). The other Aβ isoforms reproducibly detected in all spectra (Aβ1-17, Aβ1-18, Aβ1-19, Aβ1-33, Aβ1-37, Aβ1-38, Aβ1-40, and Aβ1-42) did not differ significantly in intensity before and after treatment. The levels of the Aβ isoforms Aβ1-13 and Aβ1-20 were low, were not detected in all samples, and were therefore omitted from further analysis. The mass-to-charge (m/z) ratio of Aβ1-18 [M + H]^+ ^is almost identical to the m/z of the second charge state of Aβ1-40 [M + 2H]^2+ ^and therefore difficult to quantify; it was excluded from further analysis.

In the 100-mg group, after drug administration at week 14, the CSF levels of Aβ1-14, Aβ1-15, and Aβ1-16 were significantly increased by a mean of 57%, 21%, and 30%, respectively, whereas Aβ1-34 decreased 39% (see Figure 3b and Table 2) compared with study baseline. The same result was seen in the 140-mg group, Aβ1-14, Aβ1-15 and Aβ1-16 were increased by means of 74%, 35%, and 67%, respectively, whereas Aβ1-34 decreased 37% (Table 2). No statistically significant changes were seen for any other Aβ isoforms detected. α-sAPP showed a tendency to increased levels (13%) in the 140-mg group, whereas β-sAPP was unaffected by all the treatments (Figure 3c).

**Table 2:** Summary of Mann-Whitney test for peptides

	Placebo vs. 100 mg	Placebo vs. 140 mg	100 mg vs. 140 mg
Aβ1-14	0.002	0.03	0.58
Aβ1-15	0.04	0.004	0.17
Aβ1-16	0.05	0.003	0.04
Aβ1-34	0.00005	0.0002	0.82

Kruskal-Wallace test, *P *< 0.05.

## Discussion

Intense multidisciplinary research efforts during the last decades have provided detailed knowledge on the molecular pathogenesis of AD, which has been translated into novel promising therapies with putative disease-modifying effects. Several promising drug candidates, such as Aβ immunotherapy and secretase inhibitors, are now being tested in clinical trials. However, because the predicted clinical effect of this type of disease-modifying drugs is a less-pronounced slope in the rate of cognitive deterioration, without any early symptomatologic effect, very large clinical trials with extended treatment periods will be needed to identify a beneficial clinical effect by using rating scales. Thus, biomarker evidence from smaller short-term clinical trials that the drug has the predicted biochemical mode of action directly in patients with AD would be valuable for making a go/no-go decision for expensive Phase III clinical trials. Thus, a great need exists for biomarkers to identify and monitor the biochemical effect of disease-modifying drugs in AD clinical trials.

The main focus with disease-modifying drugs is to inhibit brain Aβ production and aggregation and to increase Aβ clearance from the brain. γ-Secretase inhibitors have previously been shown to reduce Aβ1-40 and Aβ1-42 production and secretion in cells and to reduce soluble Aβ and amyloid plaque burden in mice [10,15-18,23]. These results have made the γ-secretase complex one of the top targets for developing AD therapeutics. Here we show that the novel Aβ isoforms Aβ1-14, Aβ1-15, and Aβ1-16, together with Aβ1-34, may serve as sensitive biomarkers for γ-secretase inhibition by LY450139 in the CNS of AD patients.

In a previous study using ELISA measurements of CSF Aβ1-40 and Aβ1-42, the expected reduction of the peptides in response to LY450139 treatment was not found [19]. It was suggested that this lack of changes might be the result of a rapid transport of Aβ from CSF into plasma or that longer treatment duration may be required to identify changes [19]. In another study using the SILK method to examine whether an effect on Aβ production could be identified with LY450139 treatment, it was shown that Aβ production in the CNS decreased while Aβ clearance remained stable [21]. The lack of effect on CSF Aβ1-40 and Aβ1-42, despite the reduced Aβ production, may be because the different techniques are measuring different targets. SILK analysis of Aβ turnover requires that all Aβ isoforms are digested with trypsin before analysis, and a cleavage product consisting of Aβ17-28 is then measured by using MS [20]. Thus, total Aβ (that is, the mean of all of the very different Aβ isoforms that contain the Aβ17-28 sequence) is measured. This means that all longer isoforms detected in the present study (Aβ1-33, Aβ1-34, Aβ1-37, Aβ1-38, Aβ1-40, and Aβ1-42) will contribute to the mass spectrometric signal. The results presented herein suggest that the reduction in Aβ1-34, the generation of which is γ-secretase dependent [10,11,27], may contribute to the overall reduction of Aβ detected in CSF by using the SILK method.

Previous experimental studies on certain cultured cells expressing wild-type human APP have shown that γ-secretase inhibitor can increase in α- and β-secretase cleavage products along with the expected increase in APP C-terminal fragments (C99 APP-CTF) [28]. Further, recent data have shown that the levels of Aβ1-14, Aβ1-15, and Aβ1-16 are elevated in cell media and CSF from transgenic mice treated with γ-secretase inhibitors and that these shorter isoforms are derived from concerted cleavages of APP by β- and α-secretase, thus reflecting a third metabolic pathway for APP [23,24]. Data in this study suggest that these shorter Aβ isoforms may be sensitive novel biomarkers for γ-secretase inhibitor treatment, even at doses that do no affect the CSF levels of Aβ1-40 or Aβ1-42. The increased levels of the isoforms detected after γ-secretase inhibitor treatment might be explained by an increased amount of substrate (that is, β-CTF or C99) (Figure 1), for α-secretase, after APP is cleaved by β-secretase.

Here we verify these findings directly in living AD patients (that is, a major increase of the shorter isoforms and a slight increase of α-sAPP, in CSF from AD patients treated with a γ-secretase inhibitor). The unchanged CSF levels of Aβ1-40 and Aβ1-42 are in agreement with previous studies [19] and suggest that these markers are less sensitive to detect γ-secretase inhibition as compared with Aβ1-14, Aβ1-15, and Aβ1-16, possibly because they may be influenced by other factors, such as brain amyloid load and/or Aβ oligomerization or that they are present at higher levels in CSF and that higher doses are needed to detect effects on these biomarkers in AD patients. Unchanged β-sAPP levels and only slightly elevated α-sAPP levels suggest that γ-secretase inhibition does not result in any major change in the release of these APP fragments to the CSF. It is at this stage unclear whether these fragments are degraded further or if they simply are present in such high concentrations in the CSF that a very high γ-secretase inhibitor dose is needed to detect an effect on CSF α-sAPP and β-sAPP levels.

## Conclusions

This study suggests that Aβ1-14, Aβ1-15, and Aβ1-16 are positive and very sensitive biomarkers for γ-secretase inhibition and can be used to detect biochemical effects on APP processing in AD patients treated with LY450139. Long-term clinical trials are needed to reveal whether these biomarkers predict a beneficial clinical treatment effect.

## Abbreviations

Aβ: Amyloid β; AD: Alzheimer disease; APP: amyloid precursor protein; BACE1: β-site APP-cleaving enzyme 1 gene; CNS: central nervous system; CSF: cerebrospinal fluid; ELISA: enzyme-linked immunosorbent assay; IP-MS: immunoprecipitation mass spectrometry; MALDI-TOF: matrix-assisted laser desorption/ionization time-of-flight; NINCDS-ADRDA: Institute of Neurological and Communicative Disorders and Stroke and the Alzheimer Disease and Related Disorders Association; PS: presenilin; α-sAPP: α-secretase cleaved soluble APP; β-sAPP: β-secretase cleaved soluble APP; SILK: stable isotope-labeling kinetic.

## Competing interests

The clinical trial part of the study was sponsored by Eli Lilly & Company. For the biochemistry part, the sponsors had no role in study design, data collection, data analysis, data interpretation, or writing of the article. Dr. Erik Portelius, BSc, Mikael Gustavsson, Dr. Ulf Andreasson, and Henrik Zetterberg, MD, declare that they have no competing interests. Kaj Blennow, MD, has on one occasion served on an advisory board for Eli Lilly & Company. Robert A. Dean, MD, and Eric Siemers, MD, are employees of Eli Lilly & Company and therefore are stockholders.

## Authors' contributions

EP planned the experimental design, analyzed and interpreted data, and drafted the manuscript. RAD designed the clinical trial. MG acquired data. UA analyzed and interpreted data. HZ analyzed and interpreted data. ES designed the clinical trial. KB planned the experimental design and analyzed and interpreted data. All authors reviewed, revised, and agreed on the submitted version.
